# Effects of Levothyroxine Replacement Therapy on Parameters of Metabolic Syndrome and Atherosclerosis in Hypothyroid Patients: A Prospective Pilot Study

**DOI:** 10.1155/2015/147070

**Published:** 2015-03-02

**Authors:** Zoran Gluvic, Emina Sudar, Jelena Tica, Aleksandra Jovanovic, Sonja Zafirovic, Ratko Tomasevic, Esma R. Isenovic

**Affiliations:** ^1^Zemun Clinical Hospital, Vukova 9, 11080 Belgrade, Serbia; ^2^Vinca Institute of Nuclear Sciences, University of Belgrade, Laboratory of Radiobiology and Molecular Genetics, P.O. Box 522, Mike Petrovica Alasa 12-14, 11001 Belgrade, Serbia

## Abstract

The aim of this study was to investigate the effect of levothyroxine (LT4) replacement therapy during three months on some parameters of metabolic syndrome and atherosclerosis in patients with increased thyroid-stimulating hormone (TSH) level. This study included a group of 30 female patients with TSH level >4 mIU/L and 15 matched healthy controls. Intima media complex thickness (IMCT) and peak systolic flow velocity (PSFV) of superficial femoral artery were determined by Color Doppler scan. In hypothyroid subjects, BMI, SBP, DBP, and TSH were significantly increased versus controls and decreased after LT4 administration. FT4 was significantly lower in hypothyroid subjects compared with controls and significantly higher by treatment. TC, Tg, HDL-C, and LDL-C were similar to controls at baseline but TC and LDL-C were significantly decreased by LH4 treatment. IMCT was significantly increased versus controls at baseline and significantly reduced by treatment. PSFV was similar to controls at baseline and significantly decreased on treatment. In this study, we have demonstrated the effects of LT4 replacement therapy during three months of treatment on correction of risk factors of metabolic syndrome and atherosclerosis.

## 1. Introduction

Hypothyroidism is a clinical syndrome caused by thyroid hormone (TH) deficiency, due to reduced production, deranged distribution, or lack of TH effects [1, 2]. Hypothyroidism is characterized by decreased metabolic rate and by a serum thyroid-stimulating hormone (TSH) above the upper reference limit [3].

The most common form of TH replacement therapy is synthetic T4 hormone, which is generally known as levothyroxine (LT4) and it is used to suppress TSH. While some experts highlight benefits of LT4 replacement therapy, others point out the harmful effects of the above mentioned therapy [4–7].

According to the severity, hypothyroidism is divided into severe or clinical and mild or subclinical hypothyroidism (SH). SH represents a condition of mild to moderate thyroid failure characterized by normal levels of TH with mildly elevated TSH concentrations, with or without clinical symptoms [8–10]. Further, SH can be divided into two categories, depending on the magnitude of the increase in serum TSH level, with concentrations of 4.5–10 mU/L considered as a mild disease and with concentration of TSH >10 mU/L considered as a severe disease [4, 11].

Treating patients with increased TSH level, with LT4, decreases the rate of cardiovascular (CV) diseases. Some studies have not shown any benefits of LT4 replacement therapy in reducing CV morbidity and mortality [10, 12–14].

Atherosclerosis is a leading cause of mortality and morbidity in the modern world. Atherosclerosis can cause ischemic heart disease, stroke, or intermittent claudication and gangrene [15–17]. Atherosclerotic lesions commonly develop at arterial branch sites, in regions of flow disturbance. In most cases, there is a long period of silent, slowly progressive coronary atherosclerosis before these disorders become manifest. Risk factors for atherosclerosis include insulin resistance, dyslipidemia, central and visceral obesity, hypertension, endothelial dysfunction, smoking, and physical inactivity. Intima media complex thickness (IMCT) and peak systolic flow velocity (PSFV) are commonly used as a marker of atherosclerosis [18].

Hypothyroidism is associated with factors of metabolic syndrome such as dyslipidemia, hypertension, obesity, and often insulin resistance. All these factors directly contribute to accelerated atherosclerosis [19]. Some studies show association between hypothyroidism and ischemic heart disease, regardless of age, systolic blood pressure (SBP), body mass index (BMI), and total cholesterol (TC) [20], while some do not show the relationship between increased TSH and heart disease [12].

Hypothyroidism is one of the most common causes of secondary dyslipidemia [21]. It has been reported that 95% of newly diagnosed hypothyroid patients have increased level of cholesterol [13, 22–24] and 5% of hypothyroid patients have hypertriglyceridemia. Hypothyroidism leads to a decreased level of the low density lipoproteins (LDL) receptor expression on fibroblasts and hepatocytes, decreased LDL-cholesterol (LDL-C) uptake, and consequent increase in serum LDL-C levels [13, 23].

The relationship between hypothyroidism and atherosclerosis has been confirmed [25–27]. Understanding the effects of hypothyroidism on morphological and hemodynamic parameters of the functional status of blood vessels, as an indicator of early atherosclerosis, is of great importance in terms of timely introduction of LT4 replacement therapy. Thus, the aim of this study was to investigate a possible correlation between some parameters of metabolic syndrome and atherosclerosis with increased TSH and to investigate the effect of LT4 replacement therapy on lipid profile, IMCT, and PSFV in patients diagnosed with hypothyroidism.

## 2. Material and Methods

### 2.1. Subjects and Method

This study was performed in the Department of Endocrinology, Diabetes and Metabolic Disorders of Clinical Center (KBC) Zemun, Serbia, during 2009. The study included 45 female subjects, divided into two groups: a group of patients (30 subjects) with newly diagnosed hypothyroidism, with TSH level >4 mIU/L, labeled as HypoT, and a control group labeled as control, consisting of 15 euthyroid subjects, age and gender matched with the patient group. Control subjects were healthy volunteer blood donors, recruited after physical exam, with no history of any disease. None of the controls were taking any drugs affecting the levels of serum TH and lipid levels or the acceleration of atherosclerosis. From each subject, multiple serum samples were obtained after an overnight fast. The study was approved by the Local Ethics Committee of the Clinical Center Zemun and informed consent was obtained from all subjects who participated in this study.

Initially, at the time of the detection of thyroid dysfunction in the study group (HypoT group) and the consequent initiation of LT4 treatment, parameters of the metabolic syndrome (BMI, SBP, diastolic blood pressure (DBP), and lipid levels) were assessed. Subjects were subjected to Color Doppler scan of the lower-limbs blood vessels, right superficial femoral artery (SFA), in order to determine morphological and hemodynamic parameters: the IMCT and PSFV. Dose of LT4 was calculated according to the body mass, less than 1 *μ*g/kg BM for subjects with TSH levels <10 mIU/mL (average dose 50 (12,5–75) *μ*g) or 1–1,5 *μ*g/kg BM for subjects with TSH levels >10 mIU/mL (average dose 75 (50–150) *μ*g). In the cases of higher calculated doses, LT4 was gradually increased in weekly intervals. Treated subjects who did not attain reference TSH value during 3 months of treatment were excluded from the study (neglected number). After three months of LT4 substitution therapy and established laboratory euthyroidism (labeled as LT4 group), to all patients the same hormonal and metabolic parameters were assessed.

### 2.2. Anthropometric and Clinical Measurements

Body mass index (BMI) was calculated as a body mass (kg) divided by the square of their height (m^2^). Body mass measurements were performed using calibrated beam-type balance with the subject wearing light indoor clothes and no shoes and recorded to the nearest 0.1 kg. Body height was measured using Harpenden Anthropometer (Holtain Ltd., Croswell, UK). Values for SBP and DBP were obtained using the same sphygmomanometer (HS 201C1 Palm Type Sphygmomanometer, Wenzhou Hongshun Industries and Trade Co.), a standard mechanical pressure gauge, measuring on the left upper arm standard procedure. The same person performed the same procedure to each subject: triple measurement with intervals of 10 minutes. Values are expressed in millimeters of mercury (mmHg). From obtained values, mean value was calculated and furthermore statistically used.

### 2.3. Determination of Serum TSH and FT4 Concentrations

Measurements of TH concentrations were carried out in biochemical laboratory of KBC Zemun. Levels of TSH and free thyroxine (FT4) in serum were determined by Immulite 2000 [28]. Immulite method is a chemiluminescent enzyme immunometric assay, where serum sample and a ligand-labeled tracer are added to a test unit containing a polystyrene bead coated with an antibody specific to the analyte to be measured. After incubation, the test samples underwent a washing step; an antiligand enzyme is then introduced and the test samples underwent the second incubation, after which unbound enzyme was removed. Then, a substrate is added, which in the presence of the enzyme produces emission of photons, measured by the Immulite instrument, and converted into concentration. Reference values for TSH and FT4 were 0.4–4.0 mIU/L and 10–22 pmol/L, respectively.

### 2.4. Determination of Serum, Total Cholesterol (TC), Triglyceride (Tg), HDL-Cholesterol, and LDL-Cholesterol Levels

Concentrations of TC, triglyceride (Tg), and high density lipoproteins-cholesterol (HDL-C) in serum were measured on Instrumentation Laboratory autoanalyzer using enzymatic assays (Instrumentation Lab, MA, USA) [29, 30]. The values of LDL-C were calculated using Friedewald's equation [31] (LDL-C = TC-HDL-C − 0,45 ∗ TG mmol/L). TC, Tg, HDL-C, and LDL-C concentrations were expressed as mmol/L. The reference values for TC, Tg, HDL-C, and LDL-C were 3.6–5.1, <1.7, >1.1, and <3.2 in mmol/L, respectively.

### 2.5. Color Doppler Imaging of Lower-Limb Arteries

Duplex Color Doppler scans of right SFA were made using the “Acuson” 7.5 MHz linear transducer. After marking the right SFA, the thickness of the IMC (normally up to 1.1 mm in carotid artery) and the PSFV (in m/s) was measured.

### 2.6. Statistical Analyses

The Statistical Package for the Social Sciences (SPSS) 12.0 (SPSS Inc., Chicago, Illinois) statistical software package was used for all statistical calculations. Data are presented as mean ± standard deviation for continuous variables. Differences between each group were analyzed by Student's *t*-test and chi-square test. Linear correlation analysis (Spearman and Pearson) was used to test correlations between changes of IMCT and BMI as well as levels of TSH, FT4, SBP, DBP, TC, Tg, HDL-C, and LDL-C. A *P* < 0.05 (2-tailed) was considered significant.

## 3. Results

The clinical and metabolic parameters of hypothyroid and euthyroid control subjects are presented in Table 1. The mean age of hypothyroid subjects was not significantly different compared with euthyroid subjects.

**Figure 1:**
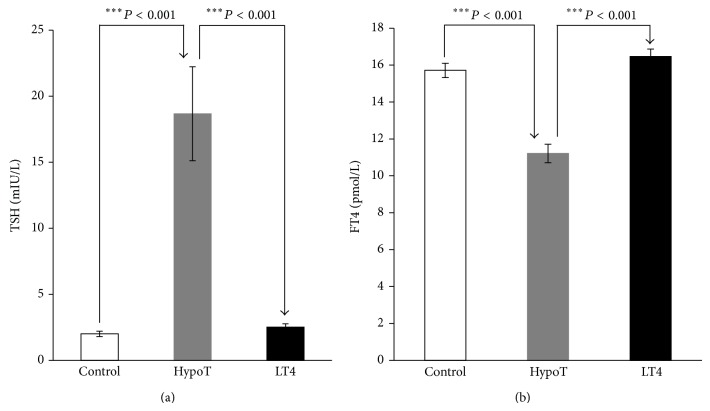
(a) Levels of thyroid-stimulating hormone (TSH) and (b) free thyroxine (FT4), in control and hypothyroid patients before (HypoT) and after treatment with LT4 (LT4). Values are given as mean ± SD.

**Figure 2:**
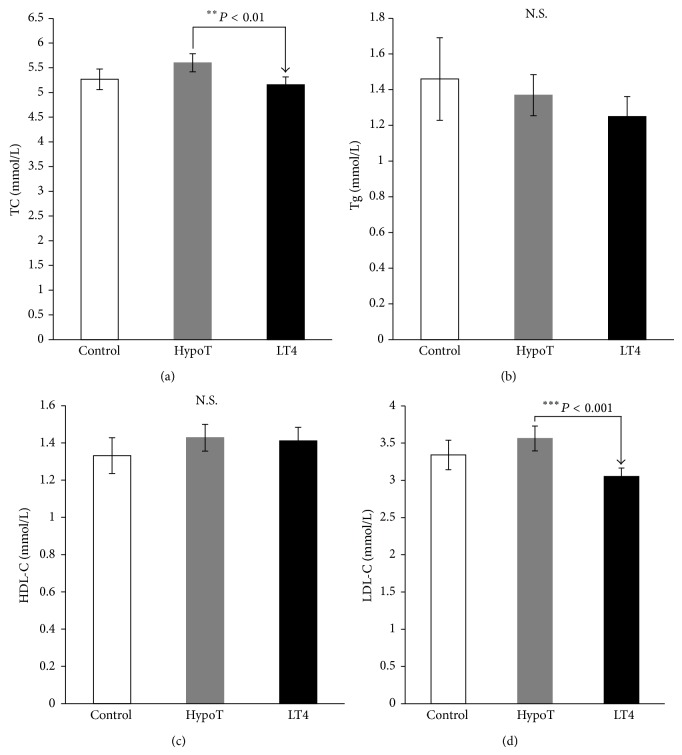
Lipid profiles: (a) total cholesterol (TC), (b) triglyceride (Tg), (c) high density lipoproteins-cholesterol (HDL-C), and (d) low density lipoproteins-cholesterol (LDL-C) of control and hypothyroid patients before (HypoT) and after treatment with LT4 (LT4). Values are given as mean ± SD. N.S.: nonsignificant.

Hypothyroid subjects have significantly higher BMI (*P* < 0.05), SBP (*P* < 0.01), and DBP (*P* < 0.05) compared with control subjects (Table 1). The level of TSH was significantly higher (*P* < 0.001) (Figure 1(a)) and the level of FT4 was significantly lower (*P* < 0.001) (Figure 1(b)) in hypothyroid subjects compared with control subjects. No significant changes in TC, Tg, HDL-C, and LDL-C levels between hypothyroid and control subjects (Figure 2) were observed.

After TH replacement with LT4, in hypothyroid subjects, the values of BMI (*P* < 0.001), SBP (*P* < 0.001), and DBP (*P* < 0.001) were significantly decreased (Table 1). Furthermore, TSH level was significantly decreased (*P* < 0.001) (Figure 1(a)), and the level of FT4 was significantly increased (*P* < 0.001) (Figure 1(b)) in LT4-treated patients compared with hypothyroid subjects. The concentrations of TC (*P* < 0.01) and LDL-C (*P* < 0.001) were significantly decreased, with no changes in the level of Tg and HDL-C after hormone replacement (Figure 2).

**Figure 3:**
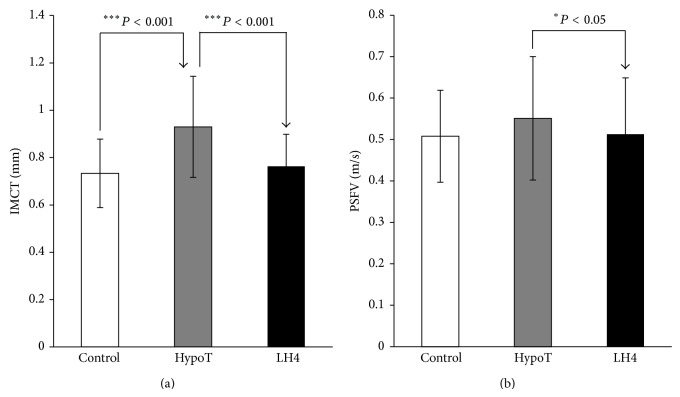
(a) Intima media complex thickness (IMCT) and (b) peak systolic flow velocity (PSFV) in control and hypothyroid patients before (HypoT) and after treatment with LT4 (LT4). Values are given as mean ± SD.

The main initial IMCT on the beginning of right SFA was significantly increased (*P* < 0.001) (Figure 3(a)) with no significant changes of PSFV (Figure 3(b)) in hypothyroid subjects compared with controls. After LT4 therapy, the values for IMCT on the beginning of right SFA (*P* < 0.001) and the PSFV (*P* < 0.05) were significantly decreased compared with the values before hormone treatment (Figures 3(a) and 3(b)).

**Figure 4:**
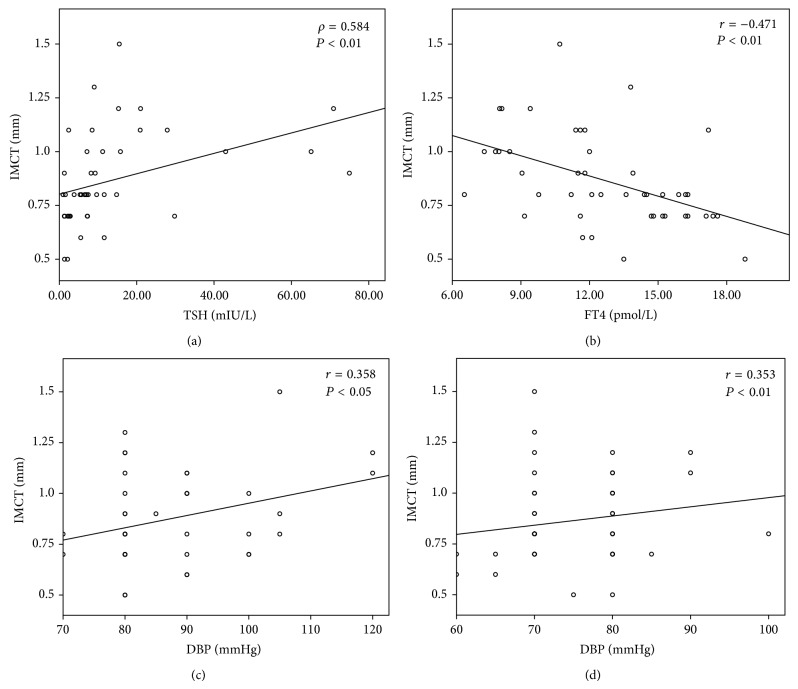
Correlation analyses between intima media complex thickness (IMCT) and (a) thyroid-stimulating hormone (TSH), (b) free thyroxine (FT4), and (c) diastolic blood pressure (DBP) before and (d) DBP after LT4 replacement treatment; *r* indicates Pearson correlation coefficient; *ρ* indicates Spearman rank correlation.

Results obtained by linear relationship analysis between IMCT and BMI, as well as levels of TSH, FT4, SBP, DBP, Tg, TC, HDL-C, and LDL-C before and after LT4 replacement treatment, show statistically significant correlations between IMCT and TSH (*ρ* = 0.584; *P* < 0.01) (Figure 4(a)), FT4 (*r* = −0.471; *P* < 0.01) (Figure 4(b)), and DBP before (*r* = 0.358; *P* < 0.05) (Figure 4(c)) and IMCT and DBP after LT4 replacement treatment (Figure 4(d)).

## 4. Discussion

In this study we have demonstrated the effects of LT4 replacement therapy during three months of treatment, on correction of risk factors for metabolic syndrome and atherosclerosis. Our results show significant effects of LT4 administration on BMI, SBP, DBP, TSH, FT4, TC, LDL-C, IMCT, and PSFV, with no significant changes of Tg and HDL-C in hypothyroid patients. In our study, patients with newly diagnosed hypothyroidism have significantly different BMI, DBP, SBP (Table 1), TSH, FT4 (Figure 1), and IMCT (Figure 3(a)), with no significant difference in lipid profile (Figure 2) and PSFV (Figure 3(b)) compared with controls. Nonsignificant difference between hypothyroid and control groups in relation to the lipid profile could be explained by the relatively high levels of lipids in the group of control subjects.

We did not observe a correlation between TSH and FT4 and lipid parameters (Tg, TC, HDL-C, and LDL-C) and PSFV. On the contrary, we have observed a significant positive correlation of TSH and a negative correlation between FT4 levels and the IMCT in hypothyroid group before LT4 treatment (Figure 4). Significant correlation between TSH level and lipid profile was not found probably due to some other parameters, that is, age and diet effects on lipid levels [32, 33]. Another possibility could be because of small sample size and small effect size. In addition, there were no significant correlations between BMI, lipid, TSH, and FT4 and PSFV. BMI and lipid profile have no significant effect on IMCT, and this could be explained by the statistical homogeneity of lipids between the groups. Our results also show the significant positive effect of DBP on IMCT (Figure 4), while BMI has no effect on IMCT.

In our study, we did not investigate the etiology of the hypothyroidism, since it is well known that Hashimoto's thyroiditis is the most common cause of increased TSH level. Stamatelopoulos et al. [34] reported that Hashimoto's thyroiditis is associated with an increased carotid-femoral PSFV independent of arterial atheromatosis indicating a direct effect on arterial stiffness. It is possible that hemodynamic consequences occurred firstly due to arterial stiffening which was followed by thickening of the IMCT. Jorde et al. suggest that the thickening of IMCT in patients with thyroid hypofunction is an early feature of atherosclerosis and that healthy controls show no significant correlation between IMCT and TSH [35]. Studies in a large Japanese population show association between thyroid function and IMCT in euthyroid individuals too [36]. Furthermore, in euthyroid persons IMCT is associated with FT4 after control of clinical factors, lipid levels, and thyroid autoantibodies [37].

The increase in TSH may be associated with an increased morbidity from CV disease and decrease of myocardial contractility [12]. The importance of treating hypothyroidism includes the correction of patients' lipid profile [38], too. A positive effect of thyroid substitution on the level of lipid fractions and reduced risk of coronary heart disease is shown in hypothyroid patients [39–43]. Thyroid function is reversible after LT4 treatment. Postmortem and epidemiological data show an increase in vascular risk in treated hypothyroid patients [44]. CV changes are reversible after euthyroid state is reached. Early initiation of the treatment of SH with LT4 can reduce cholesterol level but also may lead to development or worsening of existing coronary artery disease or to arrhythmias [12]. Some studies indicate the absence of lipid correction after substitution therapy with LT4 [41, 45].

Our results indicate a significant correction of atherosclerosis risk factors after three months of therapy with LT4. After TH replacement in hypothyroid subjects the values for BMI, SBP, DBP (Table 1), TC, and LDL-C (Figure 2) were significantly decreased compared with the values before treatment. Similar results are reported after six months of LT4 treatment, when significant reductions of TC, LDL, and IMCT (11%) in the carotid artery are reported [46]. The degree of improvement in lipid levels after biochemical euthyroidism is achieved with LT4 therapy and depends on the severity and duration of the thyroid dysfunction as well as the degree of hypercholesterolemia before the treatment [12, 24, 47]. In addition, diet, initial BMI, and smoking habits primarily could affect the level of LDL-C [48]. When the level of TSH was higher than 10 mIU/L, replacement therapy with LT4 reduced the levels of TC and LDL-C [13, 24].

More controversial is the impact of treatment with LT4 on HDL-C in hypothyroid patients. Some studies show an increase in HDL-C levels after LT4 treatment [41, 49] and others show a decrease [50, 51], whereas some studies show absence of the influence of substitutions on HDL-C level [45, 52]. Our results show that Tg and HDL-C levels were not significantly changed after hormone replacement in hypothyroid subjects (Figure 2). Meta-analysis of 13 studies shows that there was no effect of substitution on the level of HDL-C and Tg [13]. In SH patients, application of LT4 substitution caused a decline in TC (in 11 of 13 studies), while in 7 of the 9 studies there was a decrease in LDL-C [53]. These results support the hypothesis that treatment of SH is due to the favorable effect of substitution on lipid status, thus reducing the risk of coronary heart disease [12, 13, 53]. Still it is not clear to what degree treatment of SH reduces CV morbidity [53]. LT4 treatment usually corrects dyslipidemia, but if not, it is most likely that primary hyperlipidemia is associated with elevated TSH level [22].

After TH replacement in hypothyroid subjects the values for IMCT (Figure 3(a)) and PSFV (Figure 3(b)) were significantly decreased compared with the values before treatment with hormone. A significant decrease in IMCT is probably accompanied with the fall of TC and TSH. Thus, the arterial wall changes occur early with increase of TSH and LT4 treatment improving the lipid profile and decreasing IMCT. Taken together, these results suggest that the lipid infiltration of the arterial wall may be the principle mechanism of IMCT thickening [46].

In conclusion, we reported significant metabolic effects of LT4 replacement therapy in hypothyroid patients. Our study is the first to examine the effects of LT4 on factors of metabolic syndrome and atherosclerosis. The improvement of IMCT in hypothyroid patients after LT4 therapy could contribute to reduced CV risk in this patient population.

The limitation of our study is a small number of hypothyroid and healthy subjects. Given that our study included a small number of subjects, we are not able to completely show the effects of replacement therapy. Future studies on a larger population size are needed to elucidate the effects of hypothyroidism on IMCT and PSFV. This is the first phase of a prospective plot study to examine the effects on LT4 therapy on lipid and hemodynamic and morphological parameters, in Serbian population.

## Figures and Tables

**Table 1:** Basic clinical parameters in control and hypothyroid patients before (HypoT) and after treatment with LT4 (LT4).

Basic clinical parameters	Study groups			*P* value (*t*-test)
	Control	HypoT	LT4	
Age [year] [*X* ± SD (min–max)]	44 ± 10 (29–61)	49 ± 10 (28–64)	49 ± 10 (28–64)	N.S.

BMI [kg/m^2^] [*X* ± SD (min–max)]	25.65 ± 2.9 (21.5–38.9)	28.47 ± 4.1 (21.6–37.9)	27.68 ± 4.0 (20.2–36.4)	<0.05^*^ <0.001^###^

SBP [mmHg] [*X* ± SD (min–max)]	121 ± 14 (100–150)	138 ± 25 (100–210)	118 ± 17 (90–170)	<0.01^**^ <0.001^###^

DBP [mmHg] [*X* ± SD (min–max)]	80 ± 10 (70–100)	88 ± 13 (70–120)	74 ± 9 (60–100)	<0.05^*^ <0.001^###^

BMI: body mass index, SBP: systolic blood pressure, DBP: diastolic blood pressure, HypoT: hypothyroid patients, and LT4: patients after treatment with LT4. Values are given as mean ± SD; ^*^ *P* < 0.05; ^**^ *P* < 0.01. ∗ represents significance between control and hypothyroid patients before treatment; ^###^ *P* < 0.001. # represents significance between hypothyroid patients before and after treatment.

## Abbreviations

SFA: Superficial femoral artery
BMI: Body mass index
CV: Cardiovascular
FT4: Free thyroxine
HDL-C: High density lipoproteins-cholesterol
IMCT: Intima media complex thickness
LDL-C: Low density lipoproteins-cholesterol
LT4: Levothyroxine
PSFV: Peak systolic flow velocity
SH: Subclinical hypothyroidism
TC: Total cholesterol
Tg: Triglyceride
TH: Thyroid hormone
TSH: Thyroid-stimulating hormone.
